# Radiotherapy-Induced Malignancies: Review of Clinical Features, Pathobiology, and Evolving Approaches for Mitigating Risk

**DOI:** 10.3389/fonc.2013.00073

**Published:** 2013-04-03

**Authors:** Steve Braunstein, Jean L. Nakamura

**Affiliations:** ^1^Department of Radiation Oncology, University of California San FranciscoSan Francisco, CA, USA

**Keywords:** radiation-induced tumors, second malignant neoplasms, cancer survivorship, complications, mutations

## Abstract

One of the most significant effects of radiation therapy on normal tissues is mutagenesis, which is the basis for radiation-induced malignancies. Radiation-induced malignancies are late complications arising after radiotherapy, increasing in frequency among survivors of both pediatric and adult cancers. Genetic backgrounds harboring germline mutations in tumor suppressor genes are recognized risk factors. Some success has been found with using genome wide association studies to identify germline polymorphisms associated with susceptibility. The insights generated by genetics, epidemiology, and the development of experimental models are defining potential strategies to offer to individuals at risk for radiation-induced malignancies. Concurrent technological efforts are developing novel radiotherapy delivery to reduce irradiation of normal tissues, and thereby, to mitigate the risk of radiation-induced malignancies. The goal of this review is to discuss epidemiologic, modeling, and radiotherapy delivery data, where these lines of research intersect and their potential impact on patient care.

## Introduction

### Radiation-induced tumors

Our understanding and application of radiation-based technologies has evolved to recognize both the therapeutic and potentially detrimental effects of radiation exposure. That radiation could interact with tissues to generate useful information (for example radiographs), as well as acute injury, was recognized relatively early. However, radiation exposure and radiotherapy specifically, also produce delayed effects on normal tissues. One of the most devastating consequences of radiation exposure is radiation-induced tumorigenesis. Although the pathogenetic mechanisms underlying radiation-induced tumorigenesis are not well-defined, studying how normal tissues can be mutagenized by radiotherapy to promote malignancies can yield important insights into cellular and tissue responses to radiation-induced injury. This review will discuss the settings in which radiation-induced tumors occur, the known risk factors for radiation-induced tumorigenesis, models developed to understand this process, and radiotherapy practice in relation to this risk.

### Atomic bomb survivors

Individuals exposed to atomic bombs were of the general population at Hiroshima and Nagasaki. Long-term follow up of atomic bomb survivors has shown that tumor development was increased in this population compared to non-irradiated individuals (Ron et al., 1994; Thompson et al., 1994; Sadamori et al., 1996). Numerous analyses of this important population have been reported over the years and it is not our goal to summarize all of these, as the exposure of this population differs substantially from the delivery of radiotherapy. However, it is worth noting some of the features of tumor development of this group, which predict radiation-induced tumorigenesis in a clinical context.

Primary brain tumors in atomic bomb survivors included meningiomas, schwannomas, and gliomas (Preston et al., 2002). Notably, many tumors were diagnosed at autopsy, particularly commonly low-grade tumors such as meningiomas and pituitary adenomas, standing in contrast to radiation-induced tumors identified in the clinical studies to follow. Dose-response analyses revealed that linear dose-response for doses between 0 and 2 Sievert (Preston et al., 2002). Interestingly, plots of tumor incidence against distance from detonation as a surrogate for radiation exposure dose showed that the incidence of meningioma cases decreased significantly with increased distance (Sadamori et al., 1996). This radiation dose dependence observed in some solid tumors is recapitulated in the radiotherapy setting.

### Radiation-induced tumors after radiotherapy for non-oncologic diseases

Early clinical applications for radiotherapy included a variety of benign conditions, for example rheumatologic, dermatologic, and infectious diseases. This is an important context in which late radiation effects could be identified because, in contrast to malignant diseases, the long survival of these patients accommodated the long latency of radiation-induced tumorigenesis. This latency is a hallmark of radiation-induced tumorigenesis, which was first formally described by Cahan et al. (1948) in the 1940s. This classic study describes a series of patients who received radiation therapy for bone cysts, and after a latency of 5 or more years, then developed in-field malignancies such as osteosarcomas.

Low dose irradiation given over a few fractions has been used in the past for diverse conditions such as tinea capitis (Modan et al., 1974), acne (Albright and Allday, 1967), tonsillar hyperplasia, hemangioma (Li et al., 1974), and ankylosing spondylitis (Smith and Doll, 1982), resulting in the initiation of solid or hematologic tumors. For example, low dose radiotherapy for ankylosing spondylitis resulted in a significantly increased rate of death from leukemias (Smith and Doll, 1982). Late radiation-induced malignancies led to the abandonment of radiotherapy in the management of these benign conditions. However, these early experiences are worth reviewing because they provide substantial information defining the relationship of radiation dose to tumor risk in the general population, and such radiation dosing is unlikely to be replicated in any modern clinical context.

The use of radiotherapy for tinea capitis led to the irradiation of thousands of children receiving low dose, superficially directed irradiation to the scalp, with lead shielding of the face and neck typically employed (Ron et al., 1988a,b). These treatments involved low energies of 100 kVp or less, intended to deposit dose at superficial depths, and employed doses ranging from 1.0 to 6.0 Gy generally delivered in a single fraction, resulting in a mean average dose to the brain of 1.5 Gy (Ron et al., 1988b). Long-term follow up revealed that these treatments were associated with a significant risk in the development of tumors in the head and neck region and the central nervous system. The most frequently occurring brain tumors were meningiomas followed by gliomas (Ron et al., 1988b). Tumors were first noted 6 years after irradiation and continued for at least 29 years, and there was no evidence of a reduction of cancer risk toward baseline at the end of the follow up period (Ron et al., 1988b). Additional analyses of this cohort of patients estimated that statistically significant increases in risk were observed for bone and connective tissue cancers and leukemias (Ron et al., 1988a). Age at the time of irradiation also appeared to influence risk of radiation associated neoplasms, with children over the age of 10 at the time of irradiation at lowest risk, and those irradiated between 5 and 9 years of age being at highest risk of developing leukemias and head and neck cancers (Ron et al., 1988a).

These unique early data from low dose radiotherapy establish central features of radiation-induced tumorigenesis that are further developed in the setting of modern radiotherapy. These features are: (1) radiation-induced tumorigenesis occurs at low dose levels and risk increases with dose, (2) the latency of tumor detection is typically several years and can extend for decades, and (3) young age at the time of exposure is a risk factor for tumorigenesis.

## Second Malignant Neoplasms

Second malignant neoplasms (SMNs) are late complications arising after exposure to genotoxic therapies, which include radiotherapy and some chemotherapeutic agents (Neglia et al., 1991). SMNs comprise a significant fraction of subsequent malignancies in cancer survivors (Table 1). SMNs account for most of the ∼90,000 s cancers diagnosed annually in the United States (Bhatia and Sklar, 2002), and are a significant and growing late complication in survivors (Guibout et al., 2005; Henderson et al., 2007; Armstrong et al., 2009a,b; Laverdiere et al., 2009; Meadows et al., 2009; Breslow et al., 2010; Friedman et al., 2010; Ginsberg et al., 2010; Castellino et al., 2011). Radiation-induced tumors comprise the majority of SMNs. Similar to tumorigenesis after low dose irradiation for benign diseases, SMNs also develop after a latency of several years and sometimes decades (Kleinschmidt-DeMasters and Lillehei, 1995).

**Table 1:** **Recent reports of secondary malignant neoplasms (SMNs) in select cancer populations**.

Reference	Primary malignancy	Number of patients (# rec’d RT)	Age at primary diagnosis	Years of follow up	Latency to SMN development	Cumulative incidence of SMNs	Predominant RT-related SMNs	Risk factors
Rizzo et al. (2009)	Leukemia	29,000 (20,152)	Median 27 years	Median <5 years	Median >15 years	3.3% at 20 years	CNS thyroid breast	Irradiation, particularly irradiation at ages <10 years associated with 55-fold increase in risk
Taylor et al. (2010)	Mixed: leukemia, CNS tumor	17,980 (9,223)	Median <10 years	>5 years	Mean 20.5 years	3.6% at 40 years	Meningiomas gliomas	Radiation dose, intrathecal methotrexate
Armstrong et al. (2009a)	CNS tumors	2,821 (1,569)	Median <10 years	Median 19.6 years	Median >15 years	7.1% at 25 years	CNS thyroid STS	RT dose >50 Gy assoc with 7.1% SMN risk at 25 years
Friedman et al. (2010)	Mixed histologies	14,359 (8,536)	Mean 78 years	Mean 25.5 years	Mean 19 years	20.5% at 30 years	Breast thyroid CNS	SMN risk greatest for Hodgkin’s Disease survivors
Laverdiere et al. (2009)	Neuroblastoma	954 (400)	Median <10 years	Median <20 years	Median at >20 years	7% at 30 years	Thyroid renal STS	Radiation and etoposide exsoure
Breslow et al. (2010)	Wilm’s tumor	13,351 (n/a)	Mean 3.6 years	Mean 11.6 years	Median at >20 years	6.7% at 25 years	Gastrointestinal breast leukemia	Radiation
Meadows et al. (2009)	Mixed histologies	14,363 (8,412)	Mean 8.3 years	Median >5 years	Median >20 years	9.3% at 30 years	Breast thyroid CNS	0.33 ERR/Gy for glioma; 1.06 ERR/Gy for meningioma; 1.3 ERR/Gy for thyroid ca up to 6 Gy
Guibout et al. (2005)	Mixed histologies	1,814 (1,258)	Mean 6 years	Median 16 years	Median >20 years	2.8% at 30 years	Breast	0.13 ERR/Gy
Zelefsky et al. (2012)	Prostate	1,310 (1,310)	Median >65 years	Median >84 months	Median >5 years	13% at 7 years	Colorectal bladder	Increased age; EBRT vs. BT for skin cancers
Brown et al. (2010)	Endometrial	69,739 (25,106)	Mean 62 years	Median 11.2 years	Median 9.8 years	26.2% at 30 years	Colorectal bladder hematologic	Radiation increased risk of colon, bladder, rectal cancers
Chaturvedi et al. (2007)	Cervical	104,760 (52,613)	Mean 50 years	Mean 12.2 years	Median >9 years	15% at 30 years	Colorectal bladder genital	Radiation; age <50 years at primary diagnosis

*ERR, excess relative risk; RT, radiotherapy; EBRT, external beam radiotherapy; BT, brachytherapy; Gy, gray; STS, soft tissue sarcoma*.

Given the long latency of radiation-induced tumors, this is a complication that preferentially affects cancer survivors, of which there are an estimated 12 million in the United States (Underwood et al., 2012). Radiotherapy is an important component of many cancer therapy paradigms, and diverse radiotherapy approaches are used in variable settings. In addition, radiotherapy is most commonly delivered focally, and therefore the spectrum of radiation-induced tumors largely reflects the in-field tissues. Survivors of pediatric cancers are at increased risk for developing second and even third cancers, some of which are multiple and distinct SMNs, and the reasons for this susceptibility are not well understood (Armstrong et al., 2011). Defining and managing the intrinsic, or background, cancer susceptibility of cancer patients poses multiple challenges being addressed efforts in mathematical modeling, experimental modeling, and radiation physics, as will be discussed below. We will first outline major clinical settings in which SMNs develop in order to highlight important themes in radiation-induced tumorigenesis.

### Radiotherapy for leukemia

Total body irradiation (TBI) is a standard component of bone marrow transplantation protocols, and leads to broad irradiation of multiple anatomic regions and tissue types (Hill-Kayser et al., 2011). Survivors of leukemias are at risk for developing diverse malignancies, although SMNs related to cranial irradiation (CI) represent a major fraction of SMNs (Neglia et al., 1991; Banerjee et al., 2009; Rizzo et al., 2009).

Cranial or craniospinal irradiation is a major component of leukemia therapy, typically used for high risk patients (Schmid et al., 2005). Because long-term survival from childhood leukemia has improved markedly over the last few decades (Neglia et al., 1991), considerable data concerning late toxicities of cancer therapy have been obtained from this group of patients. Data from the Children’s Cancer Study Group examined 9720 children with a diagnosis of acute lymphoblastic leukemia (ALL) most of whom received chemotherapy, radiotherapy, or both. Some of these patients received cranial or craniospinal irradiation at doses ranging from 18 to 24 Gy (Neglia et al., 1991). With a median follow up of 4.7 years, a retrospective cohort study estimated a sevenfold excess for all cancers and a 22-fold excess of tumors of the central nervous system (Neglia et al., 1991). Similar to the experience of radiotherapy for benign diseases, the risk of central nervous system tumors after irradiation was significantly higher in children 5 years of age or younger at the time of diagnosis compared to patients who were older than 5 (Neglia et al., 1991). Despite patients receiving chemotherapy, radiotherapy, and both, central nervous system tumors developed in children who had been irradiated and no association was observed with exposure to cyclophosphamide or anthracyclines (Neglia et al., 1991). The most common SMNs in these patients were tumors of the central nervous system, followed by leukemias and lymphomas. The incidence of SMNs showed no evidence of reaching a plateau 15 years after diagnosis, suggesting that the risk of SMNs after irradiation persists for an extended period of time, and possibly is life-long. While modern efforts are focused on implementing reduced-intensity conditioning regimens, the feasibility of reduced-dose TBI is still unclear (Adkins and DiPersio, 2008).

The Childhood Cancer Survivor Study (CCSS) is a multi-institutional, long-term cohort study supported by the National Cancer Institute and has performed numerous studies of late effects of cancer therapy in survivors of childhood cancers. CCSS published an important study describing a matched case-control study of 14,361 5-year survivors of cancer (Neglia et al., 2006), the largest series of cancer survivors to be assessed for SMNs. Brain tumors were significant SMNs to arise in these childhood cancer survivors, with meningiomas being the most common CNS primary tumor followed by glioma, most of these being high-grade tumors (Neglia et al., 2006). The excess relative risk of brain tumor increased with increasing dose for both these tumor types, and similar to other studies, the excess relative risk was greatest among children exposed at less than 5 years of age (Neglia et al., 2006). In this large cohort of patients gliomas occurred at a median of 9 years from the original cancer diagnosis and meningiomas a median of 17 years, with no reduction in brain tumor incidence with increased follow up. These data and data from other large studies indicate that the risk of SMNs expressed as brain tumors is sustained for decades and implies that survivors of childhood cancers face continued risk of radiation-induced tumors as older adults (Taylor et al., 2010).

Moreover, radiation-induced meningiomas are often multiple, in contrast to meningiomas unassociated with prior radiation therapy (Harrison et al., 1991). Their incidence is associated with younger age and tumors can be aggressive histologies (Elbabaa et al., 2012). As compared to sporadic meningiomas, these appear to possess distinct cytogenetics, including deletions within chromosome 1p and 22q (Brassesco et al., 2012; Elbabaa et al., 2012).

### Thoracic irradiation

Hodgkin disease (HD) is a malignancy involving lymph nodal regions and is treated with chemotherapy and radiotherapy. HD commonly involves cervical and mediastinal lymph nodes, and classic radiotherapy for Hodgkin’s disease targets these nodal regions, resulting in the irradiation of mammary tissues and lung. The classic mantle field was designed decades ago to address the nodal regions commonly involved in HD and consists of fractionated radiation directed to the cervical, supraclavicular, infraclavicular, and mediastinal lymph nodes (Koh et al., 2007). This broad nodal irradiation results in diverse normal tissues receiving radiation, with multiple late toxicities potentially developing. Consequently, survivors of HD are at risk for developing radiation-induced breast cancers (Dores et al., 2002; Aleman et al., 2003; Basu et al., 2008; Crump and Hodgson, 2009; Milano et al., 2010; Castellino et al., 2011), lung cancer (Gilbert et al., 2003), as well as thyroid cancer (Hancock et al., 1991). Notably, the risk of radiation-induced breast cancers in survivors of HD has been estimated to be similar to that of individuals with BRCA1 mutations (Travis et al., 2005a).

The risk of breast cancer after radiotherapy and chemotherapy for HD is dose-dependent, with a dose of 4 Gy or more associated with a 3.2-fold increased risk compared to patients receiving lower doses, and the risk increasing to eightfold with doses of more than 40 Gy (Travis et al., 2003). The risk of breast cancer after chemotherapy and radiotherapy appears to be primarily attributable to radiotherapy, as treatment with alkylating agents alone resulted in a reduced risk. The risk of breast cancer decreased with increasing number of alkylating agent cycles, likely reflecting the reduced use of radiotherapy in these patients (Travis et al., 2003). Similar to radiation-induced malignancies in other organs, the risk of radiation-induced breast cancers persisted for more than 25 years (Travis et al., 2003). HD develops in adolescent girls and young women, and in this group, low dose radiation to breast tissue was associated with radiation-induced tumorigenesis. Overall, this study estimated that among 1000 women treated for HD at age 30 years or younger with mantle radiation alone using 40 Gy and followed for 25 years, an excess of 83 breast cancers might be observed, which might be reduced to a excess of 21 breast cancers if radiation doses were lowered to 10 Gy (Travis et al., 2003).

Radiation-induced breast cancers can also develop after radiotherapy of primary breast cancer, when tangential irradiation leads to scatter of radiation to the contralateral breast (Hooning et al., 2008). Women with breast cancer have up to 50% increased risk of developing a secondary malignancy, which is largely attributable to cancer development in the contralateral breast. Breast radiation has been linked to high risk of lung cancer development (Rubino et al., 2002), although modern radiotherapy techniques may further minimize this risk (Inskip et al., 1994). The risk of breast cancer is influenced by hormone status, as women with histories of ovarian irradiation of 5 Gy or more had reduced risk of breast cancer compared to women who did not (Travis et al., 2003). Consistent with the hormone-dependence, radiation-induced breast cancers were significantly less likely to develop in women who were menopausal before the age of 40 (Travis et al., 2003).

### Head and neck irradiation

Radiotherapy is commonly used in the management of cancers of the head and neck region. Historical rates of radiation-related neoplasms has been estimated at 15% within 5 years of radiotherapy in treatment of head and neck cancers, most of which frequently arise in the head and neck, esophagus, or lung (Cooper et al., 1989). The high incidence of SMNs following head and neck radiotherapy may be augmented by dysplasia related to significant tobacco and alcohol exposure, which are independent risk factors for primary head and neck tumors, especially of the larynx and hypopharynx (Lubin et al., 2009).

### Genitourinary irradiation

Although radiation-induced brain and breast cancers are common SMNs in survivors of pediatric cancers, radiation-induced tumorigenesis can occur after pelvic and abdominal irradiation. Survivors of testicular cancer for example are at increased risk of developing radiation-induced tumors of the digestive and genitourinary tracts (Travis et al., 1996, 2005b; van den Belt-Dusebout et al., 2007). Similarly, survivors of cervical and endometrial cancers who receive radiotherapy are at increased risk for second cancers arising in the colon, rectum, bladder, and genital sites (Chaturvedi et al., 2007; Brown et al., 2010).

Survivors of prostate cancer are also at risk for developing radiation-induced tumors (Zelefsky et al., 2012), which is particularly interesting given that these patients are generally treated at substantially older ages than testicular or cervical cancer patients. Recent SEER analysis for men with prostate cancer treated between 1988 and 2003 demonstrated a 1.88 relative risk of secondary bladder cancer incidence for those receiving external beam radiotherapy as compared with prostatectomy (Nieder et al., 2008), although this is influenced by tobacco exposure and may be decreasing in the era of modern radiotherapy techniques (Boorjian et al., 2007).

### Hematologic malignancies as SMNs

Radiation-induced malignancies also include myeloid leukemias, which develop in both humans and mice (Major and Mole, 1978; Hijiya et al., 2009; Iwanaga et al., 2011). A case-control study in a cohort of women with cervical cancer showed that the risk of leukemia increased with increasing radiation doses of up to 4 Gy, then decreased at higher doses (Boice et al., 1987). This data is consistent with the leukemogenesis observed in other low dose settings, such as treatments of benign disease (i.e., ankylosing spondylitis) described above. Further, this dose dependence would explain the predominance of solid tumors as SMNs after modern radiotherapy, which employ much higher doses.

The development of hematologic malignancies after low dose irradiation has been postulated to reflect the unique sensitivity of bone marrow cells from which leukemias originate, with higher radiation doses killing these cells so that mutagenesis cannot be expressed as future disease.

## Modifiers of Radiation-Induced Tumorigenesis

### Genetic background

Genetic backgrounds harboring germline mutations in tumor suppressor genes are recognized risk factors for cancer in general and also SMNs (Kleinerman, 2009). Tumor predisposition syndromes highlight central molecules and pathways involved in cancer, for example p53, and dysregulated Ras and Ras effector kinase signaling [Cowden’s disease, tuberous sclerosis, and Neurofibromatosis I (NF1)]. Germline mutations in *Trp53* cause Li–Fraumeni syndrome, a cancer predisposition syndrome characterized by the propensity to develop breast cancers, brain tumors, sarcomas, and leukemias (Kemp et al., 1994; Hisada et al., 1998). Because the background tumorigenesis risk in these individuals is so high, estimates of the excess risk of cancer after radiation exposure have been difficult to develop. However, given central role of the p53 protein in DNA damage responses and cell cycle regulation, it is highly likely that exposing individuals with Li–Fraumeni syndrome to genotoxins will accelerate their risk of malignancy. Individuals developing SMNs have been found to have germline mutations in p53 (Malkin et al., 1992).

Familial retinoblastoma is probably the best characterized with regard to excess risk of cancer development after radiation exposure. Familial retinoblastoma is caused by a germline mutation in the *Rb* gene, which produces the Rb protein involved in cell cycle regulation (Sage, 2012). Familial retinoblastoma is typically responsible for bilateral retinoblastoma in contrast to sporadic retinoblastoma. A study by Wong et al. (1997) described the significantly elevated risk of second cancers in individuals with familial as compared to sporadic retinoblastoma. The cumulative incidence of second cancer at 50 years after diagnosis was 51% in familial retinoblastoma compared to 5% for sporadic retinoblastoma (Wong et al., 1997). Irradiated patients commonly developed soft tissue sarcomas, and interestingly the risk of developing a radiation-induced sarcoma was apparent at a threshold dose of 5 Gy, and increased to 10.7-fold for doses exceeding 60 Gy (Wong et al., 1997).

Germline mutations need not involve a known regulator of DNA damage response; individuals with NF1 are at increased risk of developing SMNs (Sharif et al., 2006) for unclear reasons. In general, individuals with tumor predisposition syndromes should be considered at risk for SMNs after radiation. Furthermore, polymorphisms in metabolic pathways may influence SMN predisposition by modulating repair of radiation-induced genotoxic injury (Kelly and Perentesis, 2002).

### Influence of age

Survivors of pediatric malignancies are well documented to be at risk for developing radiation-induced tumors (Neglia et al., 2006; MacArthur et al., 2007). The reasons for this susceptibility are not entirely clear, although it is postulated that genotoxic injury to stem cells, which are generally more active in children as compared to adults, may be a major mechanism for the observed difference in susceptibility. Also contributing to this difference may be the extended period of survivorship in survivors of childhood cancers.

However, there is growing awareness that survivors of adult cancers also develop radiation-induced cancers after treatment of a common malignancy such as prostate cancer (Zelefsky et al., 2012). In contrast to SMNs in children, which are initiated by genotoxin exposure, SMNs in middle-aged patients may be driven by promotion of pre-existing malignant cells (Shuryak et al., 2010). This is an interesting distinction that may suggest different strategies for SMN prevention in survivors of adult or childhood cancers.

## Pathogenesis of Radiation-Induced Tumors

The molecular processes underlying susceptibility to and the development of radiation-induced tumors are not well understood. Tumorigenesis is underpinned by genetic alterations and genomic injury is a known mechanism for radiation effects on normal tissues. Currently, large scale, high genomic resolution studies have not been performed on human radiation-induced tumors to precisely characterize the genetic alterations that promote radiation-induced tumors. However, limited genetic analyses have been performed for specific histologies, for example meningiomas (Rienstein et al., 2001; Al-Mefty et al., 2004). Copy number analysis of radiation-induced and sporadic meningiomas suggests that common tumorigenic pathways may be active in both types of tumors (Rienstein et al., 2001).

Genome wide association studies (GWAS) have had some success in identifying significant predictors of cancer susceptibility in cancer survivors (Mertens et al., 2004; Best et al., 2011). However, experimental validation is also needed to justify and optimize testing chemoprevention strategies for patients.

### Mathematical models of radiation-induced tumorigenesis

Radiation-induced tumors typically arise after long latencies, and patient-based studies of SMN risk generally require follow up information from thousands of patients to reliably detect and estimate excess cancer risk after radiotherapy. There is a strong need for models that permit accurate estimates of radiation-induced cancer risk as oncologic care, and radiotherapy specifically, evolves. Epidemiological data, particularly from atomic bomb survivors, have been analyzed to develop models to help explain how the excess relative risk of cancer is influenced by several factors and how these relationships implicate specific mechanisms of tumorigenesis. The biological process of tumorigenesis can be modeled as radiation-induced initiation, or mutagenization of normal cells that then become the seed for future malignancies. Different assumptions influence these models with a central assumption being that initiation decreases with increasing age at exposure, due to the reduced time available for malignancy to develop. This assumption may to explain the markedly higher risk of radiation-induced SMNs in survivors of childhood cancers as compared to survivors of adult cancers, and would predict that radiation-induced cancer risk decreases as a function of increasing age at exposure.

Analyses of cancer risks in atomic bomb survivors indicate that the risk of radiation-induced cancers in middle-aged individuals exposed exceeds that predicted by conventional initiation-based model. Analyses by Shuryak et al. (2010) suggest that employing a combined model considering both initiation and promotion may better estimate age-dependent risk, and that the risk of radiation-induced tumorigenesis of middle-aged individuals, which describes much of the adult cancer patient population, may in fact be significantly higher than the risk estimated by initiation-only based models. These models utilize organ-specific dose volume histogram data commonly generated in modern radiotherapy planning, and represent a uniquely radiotherapy-specific phenomenon.

Additional modeling approaches consider how dose distributions within at risk organs influences radiation-induced cancer risk (Schneider and Kaser-Hotz, 2005; Schneider et al., 2005). Because increasingly conformal radiotherapy modalities differ strikingly from the radiation dose distributions achieved with two-dimensional treatment planning, at risk organs are exposed to more variable dosing, and cancer risk estimation based on an average organ dose does not account for intra-organ effects of inhomogeneous dose deposition. The concept of organ equivalent dose has been developed to account for intra-organ dose inhomogeneity, which has greater biological consequences at high doses (Schneider et al., 2005).

Model-based estimates of radiation-induced tumorigenesis allow predictions of future effects of currently evolving radiotherapy technology, and more integrated analytical approaches may uncover important insights (Shuryak et al., 2011).

### Microenvironmental contributions to radiation-induced tumorigenesis

In addition to the directly mutagenizing effects of radiotherapy on cells giving rise to tumors, changes in microenvironments after irradiation are an important area of study and potential insight into the complex process of tumorigenesis. Transplantation studies have demonstrated that irradiated microenvironments can independently promote genomic injury in stem/progenitor cells (Monje and Palmer, 2003) and enhance the expression of a neoplastic phenotype (Barcellos-Hoff, 1998; Nguyen et al., 2011). Radiation exposure can influence the remodeling of the extracellular matrix (ECM) as well as cell–cell and cell-ECM interactions (Barcellos-Hoff, 2005).

### The bystander effect

Most radiation-induced SMNs arise as tumors arising in the irradiated region, or encompassed within the radiotherapy field (“in-field” tumors), however there is evidence that the effects of radiotherapy on non-targeted tissues can influence cell and tissue function in diverse ways (Barcellos-Hoff, 2005; Shuryak et al., 2007). The bystander effect, which has been observed after radiation and chemical exposures, refers to a setting in which untreated cells demonstrate abnormalities mimicking exposure, such as chromosomal instability after irradiation (Mothersill and Seymour, 2004). Radiation-induced signals transmitted between irradiated (in-field) cells and neighboring unirradiated cells can promote the development of persistent reactive oxygen species (ROS) in unirradiated cells (Widel et al., 2012). This mechanism may promote tumorigenesis and biophysical models have been developed describing this process (Shuryak et al., 2007). The precise mechanisms underlying the bystander effect are not well-defined, but have been postulated to involve secretable factors such as cytokines and intercellular gap junctions (Mothersill and Seymour, 2004; Mancuso et al., 2011).

### Clinically based animal models of SMNs

Clinical studies of SMNs are particularly challenging because: (1) SMNs take years to develop, and patients can be lost to follow up. This is particularly true of pediatric cancer survivors, who transfer their care as adults. (2) Cancer survivors are genetically diverse, have diverse primary tumor histologies and receive diverse therapies, complicating studies to identify variables associated with increased cancer susceptibility. Mouse models are potentially powerful tools for dissecting mechanisms of human disease, but murine studies of radiation mutagenesis have been limited in their abilities to replicate clinical parameters. For example, murine studies of radiation-induced tumors have traditionally employed low dose TBI (less than 3 Gy/fraction) (Ullrich et al., 1987, 1996; Mao et al., 2005, 2008). This bears little resemblance to clinical practice, where most irradiated patients receive fractionated, focal, high dose irradiation (40–70 Gy) to a site of disease, and adjacent normal tissues at risk for mutagenesis receive 50–100% of the prescribed dose. Multiple studies indicate an important relationship between radiation dose and cancer risk in both cancer survivors and atomic bomb survivors (Tucker et al., 1987; De Bruin et al., 2009; Tukenova et al., 2011), with increasing doses associated with increasing risk of solid tumors. Data defining a clear dose-response for soft tissue sarcoma development in irradiated individuals with retinoblastoma (Wong et al., 1997) indicate that the risk of radiation-induced tumorigenesis is clearly influenced by both genetic background and the dosing of radiotherapy.

Building on clinical observations of susceptibility to SMNs, we developed mouse models of radiotherapy-induced tumorigenesis using *Nf1* mutant mice (Nakamura et al., 2011; Choi et al., 2012). We first modeled SMN development after CI and found that in-field solid tumor development after CI was significantly increased in the *Nf1* mutant background compared to wildtype, and that the tumor histologies closely reflected SMN histologies arising in cancer survivors (Nakamura et al., 2011). Focal radiotherapy promoted the development of both hematologic and solid tumor malignancies, and these classes of malignancies each developed in a dose-dependent manner. Paralleling the dose relationship of leukemia induction in irradiated patients, we found that hematologic and solid malignancies segregated such that the incidence of hematologic malignancies was reduced in high dose irradiation, in contrast to solid tumors (Nakamura et al., 2011). Importantly, radiation-induced tumors in our mouse models included well-described human SMN histologies such as soft tissue sarcomas, bone sarcoma, and carcinomas (Nakamura et al., 2011). These robust models are now serving as useful experimental platforms in which to study the interaction between genetic background and radiation. One example of the utility of these models is illustrated in the potential to perform comparative oncogenomic analysis.

Genomic damage induced by radiation exposure induces genetic alterations, some of which will be selected for in the process of tumorigenesis. Defining the common pathways responsible for tumorigenesis after irradiation will yield important insights into SMN pathogenesis and potentially reveal mechanisms that are pharmacologically targetable. Comprehensive genomic analysis of human SMNs has not been reported, most likely due to the scarcity of high quality SMN tissue samples. To overcome this limitation, comparative oncogenomics utilizes experimental mouse and human cancer genetics to reach fundamental understanding of important, conserved, and robust mechanisms of disease. Based on the well-established susceptibility of the *Nf1* mutant background used in our models, we assessed *NF1* status in radiation-induced breast cancers from survivors of HD (none having NF1) and found evidence of *NF1* loss of heterozygosity (Choi et al., 2012), indicating that this loss occurs in human SMNs. Additional studies are underway utilizing both human and murine radiation-induced tumors to identify and validate genes playing a pathogenetic role in SMN development. Identifying mechanisms important and common to SMN development may yield actionable targets for cancer prevention. In cancer survivors suspected to be at high risk for developing SMNs, it is currently not possible to predict with accuracy whether and in what tissues SMNs may develop. Efforts to analyze human SMNs for shared mechanisms of tumorigenesis have direct translational relevance because genetic changes could represent new biomarkers or targets for cancer prevention/therapy.

## Radiotherapy Techniques

Fractionated external beam radiotherapy is most common and is responsible for the majority of radiation-induced cancers. However, highly focal techniques have also been reported to produce radiation-induced tumors, although at much reduced frequency (Yu et al., 2000; Shamisa et al., 2001). For example, glioblastoma multiforme, a malignant primary brain tumor that can develop after fractionated radiotherapy, has been described as an SMN after Gamma Knife radiosurgery (Yu et al., 2000), indicating that SMNs can develop after high dose, high conformal, single fraction irradiation.

## Field Size

Radiation-induced malignancies are defined by regions of normal anatomy that are exposed to radiotherapy fields, a major motivation for the use of conformal radiotherapy techniques and reduction of field size is to limit normal tissue irradiation. This is especially pertinent in children, where a greater relative fraction of body tissue may be encompassed within standard radiation fields (Das et al., 1997; Mazonakis et al., 2003). In the case of radiation-induced tumors after HD radiotherapy, it has been estimated that involved field radiotherapy, which would lower normal tissue doses by excluding axillary irradiation, might reduce the 20-year excess relative risks of breast and lung cancers by 63 and 21%, respectively (Hodgson et al., 2007). In fact, decreases in-field size are associated with reduced incidence of SMNs after chest irradiation (Sasse et al., 2012).

## Consideration of Radiotherapy Treatment Modality

Radiation-induced SMN may occur in tissues adjacent to the target tumor volume, situated within high dose radiation portal, and generally characterized by sarcomatous histology (Dorr and Herrmann, 2002). Marked decrease in high dose treatment volumes has been achieved by more conformal external beam treatment technologies, brachytherapy approaches, and volume dose reduction protocols. Moreover, consistent patient immobilization and image-guided delivery techniques have further constrained planning treatment volume expansions. However, SMNs may also arise, and with much greater frequency, from low dose effects, typically yielding carcinomas (Dorr and Herrmann, 2002). This low dose complication is secondary to limitations in conventional beam delivery techniques, resulting in non-therapeutic scatter dose to tissues at distance from the primary treatment volume, which may initiate carcinogenesis as a late treatment effect.

### Intensity modulated radiation therapy

The development of modern external beam radiation delivery, characterized by a technological transition from rectangular portals, to irregular shapes with rigid collimation, to computer-controlled multileaf collimators, has enabled increasingly precise control of dose distribution to target tumor volumes (Brahme, 2001). This technology has developed in parallel with the emergence of routine utilization of CT, MRI, and PET based 3D imaging techniques as part of the treatment planning process (Photon Treatment Planning Collaborative Working Group, 1991a,b; Gregoire et al., 2007). Thus, the cotemporaneous improved resolution of disease reinforced the clinical rationale of reduced treatment volumes by means of incipient conformal radiation delivery technologies.

Intensity modulated radiation therapy (IMRT), which employs computer optimized control of photon fluence, has been idealized to augment the therapeutic window, by means of escalating the biologically effective dose yielding better tumor control probability, with minimization of normal tissue complications (Intensity Modulated Radiation Therapy Collaborative Working Group, 2001). Moreover, image-guidance has been integrated into many IMRT systems, further increasing precision by addressing inter- and intra-fraction variability in patient position and target motion during radiation treatment course (Mackie and Tome, 2008; Wu et al., 2011). A concern of IMRT has been the potential large integral whole-body dose due to scatter radiation associated with beam delivery, such that an extensive volume of susceptible normal tissue may receive carcinogenic low dose radiation (Purdy, 2008). Despite a roughly twofold decrease in leakage with dynamic multileaf collimators over static cerrobend blocks, as compared to conventional delivery, IMRT requires longer beam-on time and uses a larger number of treatment fields, thus delivering a larger number of monitor units associated with greater integral whole-body dose (Hall, 2006). Distant peripheral scatter doses may be even greater for pediatric patients, attributed to their small stature (Klein et al., 2006). Furthermore, while less widely reported, newer Tomotherapy-based IMRT may be associated with an even greater peripheral whole-body dose, possibly related to machine-specific treatment energies and geometries (Wiezorek et al., 2009).

There are several mechanisms contributing to combined scatter secondary radiation effects during IMRT delivery. Recognized factors include electron beam energy, distance from target, tissue depth, as well as multileaf collimator and gantry construction. At energies of 10 MV and above, neutrons are generated via beamline interactions with the primary collimator, jaws, electron target, and flattening filter. Whereas photons decrease exponentially with distance from primary treatment volume, neutrons are a significant contributor to out-of-field dose with a deposition pattern largely independent of distance to the target treatment field (Athar et al., 2010). In clinical practice, IMRT energies upwards of 18 MV are generally avoided due to the high relative biologic effectiveness (RBE) of neutrons and the large monitor unit requirement of IMRT (Kry et al., 2005a). There exists significant uncertainty as to the RBE of low dose high-energy neutrons for the endpoint of carcinogenesis, but current tissue-based estimates derived from A-bomb survivors and aberrant chromosome induction in peripheral blood lymphocytes values confirm higher RBE as compared to photons (Lloyd et al., 1976; Little, 1997; Preston et al., 2003; Kellerer et al., 2006). Additional studies of neutron-induced malignancies in animal models have demonstrated significant variation in tissue specific RBE estimates (Brenner and Hall, 2008). Intriguingly, while several groups suggest a small or negligible contribution of scatter and secondary neutrons to SMN risk in a variety of tissue types (Nath et al., 1984; Ruben et al., 2008), other analyses have demonstrated increased risk up to eightfold due to whole-body integral dose (Verellen and Vanhavere, 1999; Kry et al., 2005b). Despite the issue of increased scatter, IMRT is estimated to generate 285 excess fatal SMN per 10^5^ per Gy, approximately one-third less as compared to 425 for conventional photon therapy, largely attributed to greater dose reduction to the non-target volume by the primary beam (Lomax et al., 1999; Schneider et al., 2002).

### Proton therapy

The use of charged particle beams for radiation delivery has further refined dose distribution conformality of treatment volumes. Proton therapy in particular, with its characteristic Bragg peak and steep dose fall-off, has received great attention for the potential to decrease radiation-induced SMN. Protons provide excellent tumor volume dose distribution, with the added reduction of whole-body integral dose during treatment delivery, by a factor of two to three, as compared to IMRT and 3D conventional photon therapy, respectively (Lomax et al., 1999; Miralbell et al., 2002). Moreover, protons as compared to other charged particles, lack the additional low dose tail beyond the Bragg peak that is characteristic of carbon nuclei and may increase non-target dose (Jones, 2009). Carbon ion therapy, however, with its higher linear energy transfer and RBE, may offer an increased therapeutic ratio and support hypofractionation approaches (Brahme, 2004; Tsujii et al., 2004; Jones and Burnet, 2005). Several ongoing phase I/II trials are exploring the additional utility of carbon ion radiation with gliomas, hepatocellular, and rectal carcinoma as part of multimodal treatment (Combs et al., 2010, 2011, 2012).

It has been estimated that excess fatal SMN may be thus further reduced with proton therapy by two-thirds, to 158 per 10^5^, as compared to conventional photon therapy (Lomax et al., 1999; Cella et al., 2001; Schneider et al., 2002). Much of this promise, however, is attributed to active-scanned therapy over more commonly employed passive-scatter delivery techniques, which are associated with secondary neutron particle contamination. Notably, the majority of currently employed clinical proton beams utilize passive beam scattering in order to produce target-dose homogeneity. This technology introduces several components into the beam path, including scattering material, flatteners, collimators, and compensators, that result in production of high-energy secondary neutrons, as occurs with high-energy photon beam delivery (Fontenot et al., 2005). Of note, the dominant fraction of neutrons produced by proton delivery possesses energies over 100 MV, which is distinct from IMRT-based secondary neutron production. There exists limited data suggesting a geometrically higher RBE for these *very* high-energy neutrons from passively scattered proton delivery (Heimers, 1999; Mitaroff and Cern, 2002). This process may therefore significantly increase the risk of SMN due to the higher RBE neutrons as compared with other modalities (Agosteo et al., 1998; Brenner and Hall, 2008), although there is some evidence suggesting the risk may be no greater than that of scatter dose from IMRT photon therapy (Shin et al., 2009). Active-scanned therapy employs deflecting magnets to direct the proton beam within the target tumor volume, without additional modulation, obviating further interactions in the beamline path, with minimal secondary neutron production on the order of 2 mSv per treatment Gy (Schneider et al., 2002; Lomax et al., 2004). However, actively scanned beam lines require a more technologically complex setup that has hindered more widespread institution (Grozinger et al., 2006). It may be noted that proton bombardment of target tissues may produce internal secondary neutrons irrespective of delivery method that appear to most significantly contribute to secondary lung and hematologic malignancies (Schneider et al., 2002; Brenner and Hall, 2008).

As previously discussed, the clinical significance is of whole-body low-dose neutron exposure is unclear, but putatively has a high potential for carcinogenesis [Brenner and Hall, 2008; National Council on Radiation Protection and Measurements (NCRP), 1990]. Large volume exposures typical of craniospinal dosimetric models suggest an attributable lifetime risk of SMN up to 14.8% (fatal SMN risk of 5.3%), for passively scattered proton therapy (Taddei et al., 2009, 2010). Thus, it is the neutron-producing *external* scatter dose that is thought to be an “avoidable” contributor to this substantial risk of SMN (Schneider et al., 2001).

### Clinical comparison of modalities

Risk of radiation-related cancers has been widely studied in select patient groups. For cervical and prostate cancers, definitive radiation-treated cohorts may be compared with surgical controls. High dose treatment of cervical cancer was associated with increased risk of multiple secondary malignancies including bladder, rectal, vaginal, and non-Hodgkin’s lymphoma in women (Boice et al., 1988; Chaturvedi et al., 2007). Increased risk of RT-induced secondary malignancies in prostate cancer patients has been noted in rectum, bladder, esophagus, and lung, particularly for long-term survivors exceeding 10 years (Brenner et al., 2000; Schneider et al., 2006). A recent pair of treatment planning studies demonstrated a significantly diminished risk of SMN with passively scattered protons over 6-MV IMRT, for both early- and advanced-stage prostate cancer radiotherapy, respectively. While both techniques provided acceptable dose coverage to target volumes, the proton plans demonstrated lower doses at low and intermediate levels in the bladder and rectum (Chera et al., 2009; Fontenot et al., 2009). A dosimetric analysis of prostate cancer treatment from Schneider et al. (2007) further suggested the benefits of proton therapy over IMRT and 3D conformal radiotherapy (3DCRT) in dose-escalation models up to 100 Gy, with spot-scanned proton delivery associated with a 40% decreased risk of SMN as compared with 70 Gy 3DCRT.

Proton therapy has shown potential therapeutic benefit in treating adult malignancies in multiple other sites including the central nervous system and gastrointestinal tract (Allen et al., 2012). It is unclear if proton therapy offers superior outcomes in treatment of lung cancer (Grutters et al., 2010). There is cautionary evidence, however, for the necessity of appropriate image-guidance and adaptive re-planning in proton delivery, as tumor response over the treatment course may lead to greater dose deposition within proximal normal tissues associated with increased risk of acute toxicity and long-term SMN, which was not observed with comparable IMRT plans (Chang et al., 2005; Hui et al., 2008; Koay et al., 2012).

In older patients, the increased SMN risk with IMRT in long-term survivors may be ultimately justified by improved tumor control and reduced acute toxicity. However, this is less acceptable in the pediatric population. As previously noted, the improvement in cure rates for pediatric cancers has been associated with increased lifetime risk for RT-induced SMNs in survivors having received multimodal treatments that include radiation. Pediatric patients are particularly sensitive to radiation effects due to their developing organs, small stature, and potential long actuarial survival. This increased risk has been observed in studies of SMN following radiation therapy in treatment of Hodgkin’s lymphoma and testicular cancer in children and young adults (Wolden et al., 1998; Travis et al., 2005b). Thus, for pediatric patients, proton therapy has been viewed as especially potentially advantageous in reduction of RT-induced SMNs as the superior dose distribution allows for decreased integral, non-target, whole-body dose (Merchant, 2009). There is no prospective data for SMN-incidence as a primary endpoint for direct comparison of photon- vs. proton-based radiation treatment. In addition to SEER and Children’s Cancer Study Group reports, various groups have modeled SMN-incidence based on guidelines of the International Commission on Radiologic Protection or National Council on Radiation Protection and Measurements [National Research Council, 2006; International Commission on Radiological Protection (ICRP), 1991], accounting for primary target dose and secondary neutron scatter dose deposition (Schneider et al., 2000, 2001; Jiang et al., 2005; Schneider, 2005), noting the aforementioned concerns about the accuracy of the applied estimates for neutron RBE in risk models, as these are larger values extrapolated from A-bomb exposure data (Hall, 2009).

Practical estimation of the incidence of radiation-induced SMNs has been compared for IMRT vs. proton delivery plans in treatment of a variety of pediatric tumor anatomic sites and histologies. Miralbell et al. (2002) examined the advantage of proton therapy, reporting a twofold or greater reduction in SMN-incidence in a parameningeal rhabdomyosarcoma case and an 8- to 15-fold reduction in a medulloblastoma case as compared with IMRT or conventional X-ray delivery. Of note, however, this analysis neglected the contribution of stray secondary neutrons to SMN prediction, which was later addressed in a study by Newhauser et al. (2009) that demonstrated maintained, although slightly diminished, benefit of lower SMN risk with both passively scattered and scanned beam proton therapy. Another dosimetric study, by Lee et al. (2005) compared proton, IMRT, and 3DCRT modalities for several cases of pediatric retinoblastoma, medulloblastoma, and pelvic sarcoma, with protons overall demonstrating both optimal target dose coverage and normal tissue sparing, which may ultimately reduce the risk of SMN.

## Conclusion

**Figure 1:**
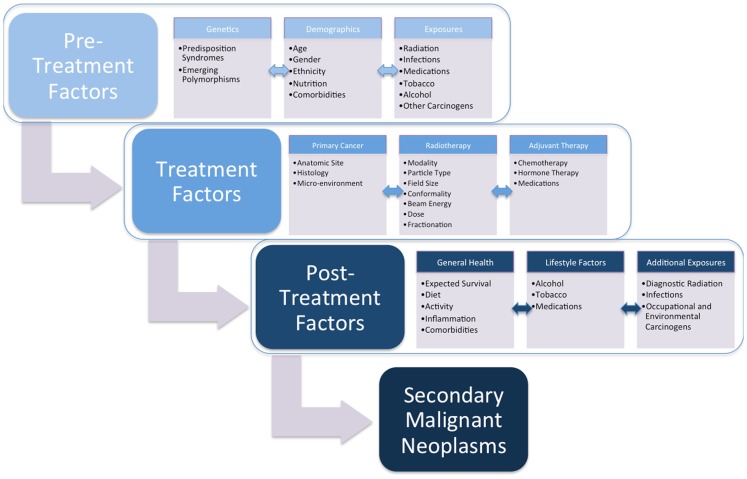
**Schematic of secondary malignant neoplasm (SMN) development**.

Radiotherapy continues to be a critical component of oncologic care. As cancer survival improves, the late effects of radiotherapy can impact long-term patient health. The most significant and life-threatening of late effects is the development of an SMN. A review of the literature demonstrates that radiation-induced tumors develop after relatively long latencies of often several years, but that this risk often persists for decades without a plateau. Defining the conditions that promote this complication will allow us to develop both treatments and cancer preventive strategies for individuals diagnosed with cancer (Figure 1). Low dose radiation is associated with an increased risk of tumor development in a variety of normal tissues, and susceptibility can be strongly influenced by genetic background and likely additional factors. These data should influence how we evaluate technologies and the care of cancer survivors moving forward.

The majority of recent studies favor of proton therapy toward a goal of reducing radiation-induced SMN, with spot-scanned delivery being of greater advantage than passive-scatter at reducing stray secondary neutrons, as compared with IMRT and 3DCRT. A major limitation in these assessments is the lack of large scale randomized controlled trials comparing late effects amongst different modalities, with SMN as a specific endpoint. In addition, there exists limited long-term secondary toxicity data for proton therapy, as presented in several institutional case-series. Much of the supporting evidence for protons is derived from theoretical comparative dosimetric models, with SMN risk assessment by various measurement techniques or Monte Carlo calculations, using generic or anthropomorphic phantom models, and with different source data for risk modeling (Chaves et al., 2004; Rodrigues et al., 2004; Fontenot et al., 2009; Taddei et al., 2009). The effects of fractionation and dose-rate are also not generally considered (Jones, 2009). Another significant concern in these comparative models is the assumption of similar tissue specific dose-effect curves independent of modality and the uncertainty of the true RBE of high-energy neutrons (Miralbell et al., 2002; Newhauser et al., 2009). While much of current SMN models are derived from A-bomb survivor data, the pattern and histologies are distinct from those observed following radiotherapy (Pierce et al., 1996; Hall, 2006). Moreover, the roles of chemotherapy, genetic-predisposing, and environmental factors are not accounted for in current models of radiation-induced SMN (Schneider et al., 2001). More recent studies have confirmed gender and age at exposure as highly significant factors in differential SMN risk with proton therapy (Armstrong et al., 2007; Zacharatou Jarlskog and Paganetti, 2008; Taddei et al., 2010). Notably, these limitations have been reflected in discrepancies between observed and predicted SMNs (Goldstein et al., 1997; Miralbell et al., 2002).

Ultimately, elevated risk of SMN remains an indelible late effect of radiation therapy, regardless of radiation modality (Hall and Wuu, 2003; Kry et al., 2005b; Brenner and Hall, 2008). Minimization of this risk is increasingly recognized as paramount, as patients are experiencing improved outcomes, associated with long-term survival, as a consequence of modern integrated treatment approaches. As recognized by the NCRP, our understanding of molecular biology and genetics must extend beyond the primary pathophysiology of presenting malignancy, in order to risk stratify patients with regard to predisposition to radiation-induced SMN, as part of individualized management (Travis et al., 2012). Irrespective of the absolute SMN risk-reduction, current clinical and theoretical evidence support proton therapy, especially in treatment of pediatric malignancies (Allen et al., 2012). New randomized trials will more conclusively evaluate the long-term clinical benefit, and thus further justify the cost of widespread and routine utilization of clinical proton technologies. In the interim, recent pediatric protocols (e.g., medulloblastoma) are applying risk-adaptive strategies that further diminish radiation extent as part of multimodal cancer management (Gajjar et al., 2006; Rutkowski et al., 2009; Ashley et al., 2012). Increasingly restricted planned treatment volumes are sought in dose-escalation for lung and prostate cancer, afforded by image-guided techniques that reduce target uncertainty (Song et al., 2005; Gomez and Chang, 2011). Brachytherapy, as demonstrated in recent prostate studies, may afford a decrease in non-target whole-body dose, with slightly reduced rates of SMN (Liauw et al., 2006; Zelefsky et al., 2012). Evolution of targeted therapies and radiosensitizers may further refine radiation exposure (Thomas et al., 2006; Shewach and Lawrence, 2007). Expectantly, such refinements in treatment approaches, concurrent with refinements in proton and other particle beam delivery technologies that further reduce “avoidable” secondary neutrons, will minimize the incidence of treatment-related SMN.

Concurrent with the continued examination of and development of technologies that minimize incidental irradiation normal tissues, efforts to define the underlying biology are also critical because (1) there is already a large population of irradiated cancer survivors for whom SMN risk is likely life-long and (2) even highly conformal radiotherapy approaches will not entirely eliminate SMN risk.

## Conflict of Interest Statement

The authors declare that the research was conducted in the absence of any commercial or financial relationships that could be construed as a potential conflict of interest.
